# MRMPath and MRMutation, Facilitating Discovery of Mass Transitions for Proteotypic Peptides in Biological Pathways Using a Bioinformatics Approach

**DOI:** 10.1155/2013/527295

**Published:** 2013-01-29

**Authors:** Chiquito Crasto, Chandrahas Narne, Mikako Kawai, Landon Wilson, Stephen Barnes

**Affiliations:** ^1^Department of Genetics, University of Alabama at Birmingham, Birmingham, AL 35294, USA; ^2^Department of Computer and Information Sciences, University of Alabama at Birmingham, Birmingham, AL 35294, USA; ^3^Department of Pharmacology and Toxicology, University of Alabama at Birmingham, Birmingham, AL 35294, USA; ^4^Centers for Nutrient-Gene Interactions, University of Alabama at Birmingham, Birmingham, AL 35294, USA; ^5^Targeted Metabolomics and Proteomics Laboratory, University of Alabama at Birmingham, Birmingham, AL 35294, USA

## Abstract

Quantitative proteomics applications in mass spectrometry depend on the knowledge of the mass-to-charge ratio (*m/z*) values of proteotypic peptides for the proteins under study and their product ions. MRMPath and MRMutation, web-based bioinformatics software that are platform independent, facilitate the recovery of this information by biologists. MRMPath utilizes publicly available information related to biological pathways in the Kyoto Encyclopedia of Genes and Genomes (KEGG) database. All the proteins involved in pathways of interest are recovered and processed *in silico* to extract information relevant to quantitative mass spectrometry analysis. Peptides may also be subjected to automated BLAST analysis to determine whether they are proteotypic. MRMutation catalogs and makes available, following processing, known (mutant) variants of proteins from the current UniProtKB database. All these results, available via the web from well-maintained, public databases, are written to an Excel spreadsheet, which the user can download and save. MRMPath and MRMutation can be freely accessed. As a system that seeks to allow two or more resources to interoperate, MRMPath represents an advance in bioinformatics tool development. As a practical matter, the MRMPath automated approach represents significant time savings to researchers.

## 1. Introduction

A feature of the last two decades of biomedical research has been the generation of “–omics” data, a result of the pursuit of *discovery*. The introduction of soft ionization techniques for analysis of peptides and proteins by mass spectrometry in the 1980s [1, 2] led to a plethora of applications related to the identification of proteins from a wide variety of proteomes, from microorganisms to plants to mammals. These studies largely defined the *measurable* peptidome and by implication the proteome. They were also designed to “discover” significant protein changes, such as abundance and modifications. Because of the complications resulting from multiple hypotheses testing, however, detecting differences between treated and control samples has often met with limited success [3]. Concern has been expressed, for example, over the failure of different participating laboratories to systematically determine the same proteins that distinguish cancer patients from controls [4].

**Figure 1:**
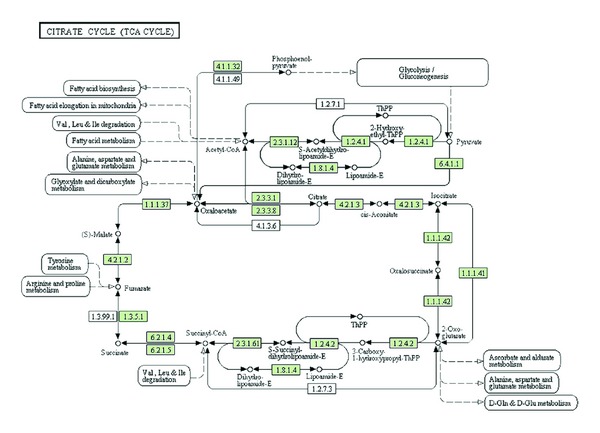
The figure represents the pathway for the citrate tricarboxylic acid cycle for humans as seen at the KEGG resource. The components, proteins, and reagents, highlighted in green, are those involved in the pathway for humans. The components not highlighted are part of the generic TCA cycle pathway. If another species is selected then only those components that contribute to the pathway are highlighted. The figure is a screen capture from the URL, http://www.genome.jp/kegg-bin/show_pathway?org_name=mmu&mapno=00062.

The next phase of proteomics is moving towards targeted, hypothesis-driven experiments. It integrates knowledge from previous proteomics discovery endeavors (2D-gel/peptide mass fingerprinting, MuDPIT, and GeLC-tandem mass spectrometry), microarray analysis (DNA, mRNA and microRNA chips, as well as DNA deep sequencing and RNA Seq), from the general scientific literature (particularly signal transduction pathways), or from the detailed study of one or several proteins in a known complex or a biological pathway.  Figure 1 represents the tricarboxylic acid (TCA) cycle for humans as visualized through the KEGG (Kyoto Encyclopedia of Genes and Genomes: http://www.KEGG.jp/).

The development of targeted analysis of proteins has been facilitated by the use of multiple reaction ion monitoring (MRM), a mass spectrometry technique commonly used for the quantitative analysis of drugs and their metabolites [5]. If trypsin is used as the protease to cleave proteins into peptides, the resulting tryptic peptides, many of which consist of seven to 25 amino acids, would be suitable for analysis by MRM-MS on a triple quadrupole mass spectrometer. The molecular ion of the tryptic peptide ion (usually doubly charged) is selected by the first quadrupole, fragmented by collision with neutral gas in the second quadrupole, and specific, peptide sequence-dependent product ions are selected by the third quadrupole. The ion intensity resulting in this double selection process is typically measured for 20–30 msec at a time. Then another molecular ion/product ion combination representing a second tryptic peptide is examined. This process can be repeated 30–50 times a second before cycling back to the molecular ion/product ion combination for the tryptic peptide for the first protein. If the signal intensities of the analytes being measured are strong enough, the period for each channel can be shortened and as many as 500 transitions a second can be monitored.

The process of ensuring that a peptide from a specific protein in a biological pathway is proteotypic, especially when done manually, is prohibitive. It involves (1) selecting protein(s) involved in a biological (disease, metabolic, etc.) pathway, clicking on the source-link for the protein(s), and accessing the web page that contains information about the protein(s), or from other sources; (2) obtaining their amino acid sequences in the FASTA format; (3) submitting the sequences for *in silico* enzymatic digestion; (4) organizing the peptides that may be suitable for analysis, (5) carrying out a BLAST search for each.

Other attempts to identify suitable tryptic peptides for quantitative LC-MRM-MS analysis have either been based on a pragmatic approach (by inspection of peptide MS/MS data collected on instruments in investigators' laboratories), or on predictive tools based on a peptide training set. The latter increases the chance of selecting high intensity peptide ions. Skyline (https://brendanx-uw1.gs.washington.edu/labkey/project/home/software/Skyline/begin.view), a downloadable tool that performs many of the above steps, is available for investigators and end-users on the Windows platform. Skyline, for a given protein or peptide, calculates the masses of tryptic peptides and their fragment ions and includes provisions to filter out oxidizable groups and to allow biochemical posttranslational modifications. While Skyline is an excellent tool for investigators with experience in the use and applications of peptide mass spectrometry, it nonetheless represents a barrier for biologists who have identified areas of biochemistry where identification of changes (by western blotting or DNA/RNA measurements) in a critical element of a pathway prompts a thorough investigation of all the components of the pathway and in some cases neighboring pathways. MRMPath and MRMutation allow the biologist to accurately recover the peptide and associated mass spectral data about the proteins in the biological pathway(s) so that the information can be transferred to the domain of mass spectrometry.

The present study therefore has two goals: (1) to create a freely-accessible, web-based software tool (MRMPath), that is, Internet browser accessible and hence not subject to dependence on the computer operating system. This software would dynamically retrieve and process known pathways of metabolism and protein signaling using an automated bioinformatics approach. Peptides that can be evaluated for their proteotypic character using BLAST searches would be extracted using this software. Any investigator would be able to access MRMPath and download and store results; and (2) the creation of a second web-based tool (MRMutation) that dynamically accesses all the known mutations (germ-line, somatic, and experimental) of a given protein in order to identify those tryptic peptides which would contain the mutation. 

## 2. Methods

The proteins associated with human diseases and other processes, including metabolic and cellular processes in many species in which the genome sequences are known, are cataloged in a publicly available web resource, the Kyoto Encyclopedia of Genes and Genomes (KEGG) (URL http://www.genome.jp/kegg/). This resource is well visited by researchers the world over. It provides information related to pathways which are categorized according to metabolic, genetic information processing, environmental information processing, cellular processes, organismal systems, and human diseases. The individual pathways include the complement of proteins that are involved in them.  Figure 1 is a screen capture, for the TCA (tricarboxylic acid) cycle in humans, presented through KEGG's web interface. The result of a user query in the KEGG for a specific pathway is a generic, non clickable representation of all the components involved in a pathway. For a pathway, a user can then choose the one appropriate for each species catalogued by KEGG. When a user chooses a species, for example, *Homo sapiens*, the proteins and other components that are specifically involved in the pathway for humans become “live” or clickable links. In Figure 1, these boxes are colored green.

For proteins and enzymes (which are represented using the Enzyme Commission nomenclature), the user is taken to a page where additional information is available related to the protein, which includes alternative nomenclature for that protein in other resources, the DNA sequence from which the protein sequence is intuitively translated, the family from which the protein arises, and the link to that protein in the FASTA format (which is accessed in MRMPath), among other information.

MRMPath facilitates the collection of the protein amino acid sequence data presented by KEGG. It is freely available on the Internet (http://tmpl.uab.edu/MRMPath/). Only an Internet browser is needed to access and use MRMPath. The system was designed for free use, mitigating the need for platform-specific computer operating systems, or to download and install software. 

**Figure 2:**
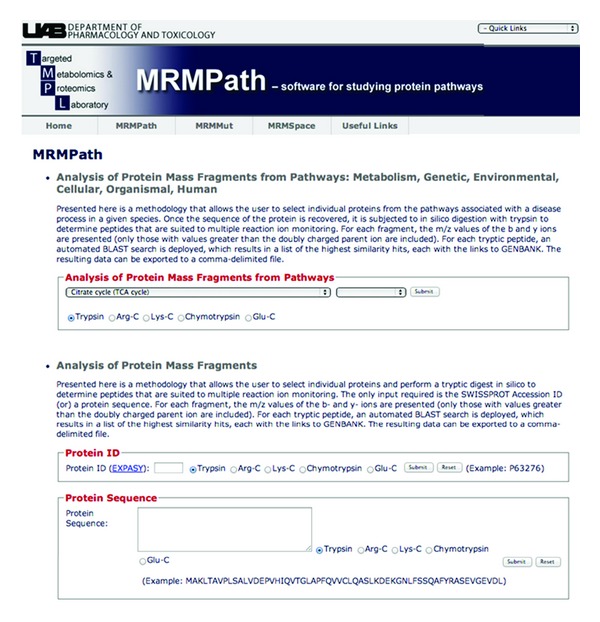
Front page of the Targeted Metabolomics and Proteomics Laboratory website. This is the home page for MRMPath and MRMutation. The three input choices for MRMPath—processing of peptides of proteins involved in biological pathways via KEGG, through Accession IDs in UniProt and direct input of a protein sequence—are illustrated.

On accessing MRMPath and clicking on the “MRMPath” link, a user is offered three choices that involve deploying MRMPath by (1) processing proteins involved in a pathway stored in KEGG; (2) processing a protein from EXPASY (formerly, SWISSPROT) by entering the EXPASY protein ID; (3) cutting and pasting the protein sequence into a text box.  Figure 2, a screen capture of the MRMPath home page, illustrates the choices that are available for protein sources.

### 2.1. MRMPath and KEGG

The first option allows users to use a drop-down menu to select among the pathways that are available in KEGG. When a user clicks on the pathway of choice, the system automatically populates a second drop-down menu which identifies only the species for which the selected pathway is available in KEGG. When the user clicks the “Submit” button, the system automatically downloads and represents the pathway to the investigator just as the user would see in KEGG, that is, with only the components (proteins) from the pathway highlighted in green as they are relevant to the species. This pathway is downloaded dynamically from the KEGG web resources and presented to the MRMPath user as a virtual webpage (precluding the need to store information); its HTML (hypertext markup language) webpage is processed and modified (on the UAB servers). The page illustrating the biological pathway for a species appears to the user exactly as it would appear to a KEGG user. The links for specific “live” components—proteins involved in the pathway for that species—are changed such that clicking on these links now deploys the MRMPath software for that protein, instead of leading to the webpage that contains additional information for that protein in KEGG.

### 2.2. MRMPath Processing

The amino acid sequence for the protein involved in the pathway is recovered in the FASTA format and subjected to *in silico* trypsin digestion (MRMPath also allows users to perform digestions using chymotrypsin, Arg-C, Lys-C, and Glu-C). Following a tryptic digest, cleavage occurs on the C-terminal side of arginine and lysine residues except when the next amino acid is proline. The mass-to-charge (*m/z*) values for the monoisotopic, doubly charged tryptic peptides are determined from the empirical formula for each peptide residue, using the elemental masses for carbon (12.00000000), hydrogen (1.00782503), nitrogen (14.00307401), and oxygen (15.99491462) [6]. Peptides with less than seven amino acids or more than 25 amino acids are not considered. Peptides containing cysteine or methionine resides are filtered out because of modifications that may arise from nonbiological events during sample processing. These peptides thus filtered are processed to calculate *m/z* values for b-ions and y-ions. Typically, these are larger than the *m/z* of the doubly charged molecular ion. In general, peptides chosen for MS/MS are doubly or triply charged; therefore, their higher mass, singly charged product ions have *m/z* values that could not arise from a singly charged molecular ion at the same *m/z* value as the doubly or triply charged peptide ion. The filtered tryptic amino acid sequences are subjected to automated BLAST analysis at NCBI (http://blast.ncbi.nlm.nih.gov/Blast.cgi). This is carried out either on a single tryptic peptide or on all the selected tryptic peptides for a given protein.

The following specific example illustrates the specific data-mining steps that the system deploys following a user query. When a user chooses a pathway and a species, MRMPath automatically creates a URL (Universal Resource Locator) which a user would otherwise manually type to access that pathway for that species. For example, consider the link http://www.genome.jp/kegg-bin/show_pathway?org_name=mmu&mapno=00062. The organism's name is identified by the three-letter species code “mmu” (*Mus musculus*). The numerical representation “0062” refers to the pathway, “fatty acid elongation.” In KEGG, this pathway is represented under the category “Metabolism,” and subcategory “Lipid Metabolism.” We initially recovered and stored the codes for all the organisms and pathways in KEGG. 

One of the components of this pathway is the mitochondrial trans-2-enoyl-CoA reductase (EC: 1.3.1.38). Within KEGG, when this component is clicked, it takes the user to information about that enzyme in KEGG through the URL (http://www.genome.jp/dbget-bin/www_bget?mmu:26922). Through MRMPath, when the pathway is downloaded and processed, each link within the downloaded file is modified. The KEGG link for mitochondrial trans-2-enoyl-CoA reductase would be automatically modified to http://www.genome.jp/dbget-bin/www_bget?-f+-n+a+mmu:26922, which is the link to the FASTA file for this protein. The FASTA formatted protein sequence is then further processed.

Leveraging pathways published at the KEGG resource is an innovative aspect of MRMPath. MRMPath can process proteins involved in pathways and makes them all available to mass spectrometry specialists.

For the two other deployment strategies available in MRMPath, the above description is the same, except that only one protein at a time is processed.

### 2.3. EXPASY

The EXPASY Bioinformatics Resource Portal (http://www.expasy.org/) contains the world's most comprehensive and highly curated repository for proteins. In addition to information related to protein sequences, this resource is constantly updated with tools and subdata bases for different aspects of the analysis of proteins. The database within EXPASY that stores and allows access to proteins is UniProtKB (http://www.uniprot.org/). This resource allows access to proteins on the database through a keyword search or a descriptor search or by using the UniProt accession ID. 

MRMPath uses web-accessibility techniques that were discussed previously to download a pathway or a FASTA-formatted protein sequence from KEGG. For example, consider a protein with a UniProt Accession ID, P47888. Clicking on the link associated with this Accession ID, http://www.uniprot.org/uniprot/P47888, allows a user to access additional information about this protein. MRMPath manipulates this link such that its algorithms can automatically access and download the FASTA formatted sequence from this protein through the webpage with the link, http://www.uniprot.org/uniprot/P47888. The sequence is thus downloaded and can be processed further by MRMPath, as described in the section on MRMPath and KEGG.

### 2.4. User Supplied Sequences

The third option, shown at the bottom of Figure 2, is a text box, which allows the user to cut and paste a protein sequence. This could be an entire protein as well as a fragment. This protein sequence is then processed through MRMPath in the same way as described when a FASTA formatted protein sequence is automatically downloaded from KEGG or from UniProt.

### 2.5. MRMPath's Process

As illustrated in the above section, the input for MRMPath is a protein or peptide sequence. This sequence can be automatically extracted for a protein involved in a biological pathway from KEGG, or a protein that is stored in the proteomics repository at EXPASY, or a user-supplied sequence. MRMPath processes a sequence as follows.

*Enzymatic Digest*. The sequence is first processed to create peptides following a theoretical enzymatic digest. MRMPath allows a user to choose between trypsin (cleaves on the carboxyl side of Arg and Lys), chymotrypsin (cleaves on the carboxyl side of Phe, Tyr, and Trp), AspN (cleaves the amino side of aspartate residues), GluC (cleaves the carboxyl side of aspartate and glutamate residues), LysC (cleaves on the carboxyl side of Lys residues), and ArgC (cleaves on the carboxyl side of Arg).

*Selectivity for Met and Cys*. The peptides obtained by the above user-determined enzymatic digest are processed to delete any that contain methionine or cysteine amino acid residues since these are susceptible to oxidation during sample processing and may not reflect the biology that is under investigation. A peptide containing methionine or cysteine may exist in several oxidized forms in addition to the unmodified peptide, rendering uncertainty in quantitative analysis.

*Selection by Peptide Sizes*. The resulting peptides are then filtered for the total number of amino acid residues. Only those peptides having seven or more, but not more than 25, residues are selected for further processing. Peptides with fewer than seven residues are unlikely to be proteotypic, whereas those with more than 25 residues become harder to detect or exceed the mass range of the quadrupole mass filter. The latter would have doubly charged ions that would be greater than *m/z* 1300.

**Figure 3:**
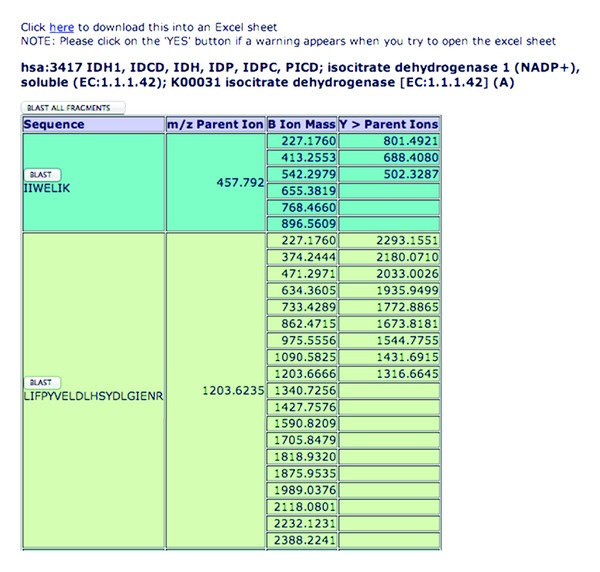
A screen capture of MRMPath results (truncated) shows the peptides for a chosen protein (isocitrate dehydrogenase) from the TCA cycle pathway. The peptides are a result of a tryptic digest, the precursor ion values and the b- and y-ions whose masses are greater than that of the precursor ion identified. The link towards the top of the page allows users to download the processing result to an Excel spreadsheet. The buttons that allow BLAST searching of individual peptides as well all peptides from the chosen protein are also illustrated in the figure.

*Theoretical MS/MS Spectrum*. For each of the resulting peptides from a protein, their multiply charged precursor ion can be collisionally dissociated producing b- and y-product ions. The y-ion masses (following cleavage at the amide bonds and containing the C-terminal residue) and b-ion masses (caused by cleaving the amide bonds and retaining from the N-terminal residue) are determined and tabulated. The *m/z* ratio for the peptide precursor ion is also calculated. These results are displayed on a browser following MRMPath's use (Figure 3).

### 2.6. BLAST Searching

**Figure 4:**
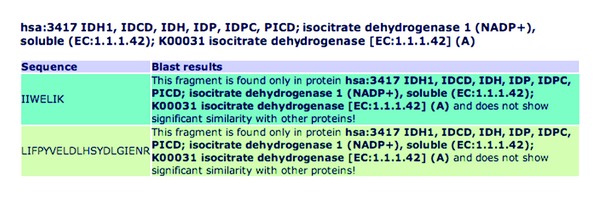
The result of a BLAST search of the tryptic peptide, the first peptide from Figure 3, shows that no results are found. The *m/z* for the parent ion of this peptide is also illustrated. If sequences similar to the peptide were identified, the top ten results would appear with links back to the GenBank resource for each similar sequence in the third column in the figure.

Figure 4 shows that a BLAST search is also included for each fragment in the results of the webpage. When a user clicks this button, an automated BLAST search is initiated. The steps involved in the BLAST search are identical to a manual BLAST search for proteins on the webpages of NCBI BLAST (http://blast.ncbi.nlm.nih.gov/). During the manual search, a user will choose the type of BLAST, protein, nucleotide, and so forth, against a specific genomics resource (RefSeq—http://www.ncbi.nlm.nih.gov/RefSeq/ e.g.). A prompt appears on the webpage indicating after how long the search is likely to be completed. The results are then presented pictorially with color codes indicating the closeness of the BLAST results. The color red indicates a high similarity, the color black indicates lower than 40 percent similarity.

In MRMPath's automated BLAST search, the process is similar to the manual web-based BLAST search (http://blast.ncbi.nlm.nih.gov/), however, without manually entering the protein sequence (using NCBI's unique identifying number for the protein, the FASTA- or free-formatted protein sequence). MRMPath's BLAST search occurs in two steps. First, following a BLAST request for a peptide, the algorithm creates a URL. Embedded into this URL are query parameters: these include the peptide sequence, number of hits to return, and data source to search (NCBI). MRMPath then scans the NCBI BLAST server to identify a Process ID created by the BLAST system and the time it will take for the BLAST search to be completed. The program then automatically suspends processing for that amount of time (this might typically last from four to five seconds, longer if the BLAST servers are busy). After this “wait” time has elapsed, the program creates a second URL which includes the Process ID and dynamically extracts the BLAST results. One difference between the automated and manual searches is that results with very small sequence similarity will not be returned in the automated system as viable results.

The top ten BLAST results are returned to the user. If strong similarities are not found, then the program informs the user that the peptide does not have significant similarity with other proteins. The top ten BLAST results (if available) are then presented to the user in a webpage, with links to the sources of the proteins in GenBank.

### 2.7. Comprehensive Processing in MRMPath

In Figure 4, at the top right hand corner is a button, “BLAST ALL FRAGMENTS.” This facility allows users to perform BLAST searches on all the peptides that result from the MRMPath filter at the same time. As has been explained previously, given that there is a wait time while BLAST searching occurs, users are likely to have to wait for several minutes for all BLAST searching on all the fragments to be complete. This process would be lengthened even more if done manually, where every fragment would have to be individually entered into the BLAST query “box.”

When a user chooses to process a protein from a pathway through KEGG, MRMPath allows the option of processing all the proteins involved in a pathway for a particular species at the same time. Each protein involved in the pathway for a user-queried species will be processed in the same way as a single protein would, user-defined enzymatic digest and selection for peptide length (7–25 amino acid residues) and peptide sequences lacking methionine and cysteine residues. 

In addition to the results being published on MRMPath's results webpage, where they can be downloaded, the results are also automatically written to an Excel spreadsheet. A new spreadsheet is generated with every MRMPath use. The same results that are available on the results web page are stored in the spreadsheet. We have placed, and continue to endeavor to place, information in the spreadsheet in the right format such that it can automatically, entered as input into the setup of manufacturer software such as Midas and MRMPilot for LC-MRM-MS analysis.

### 2.8. MRMutation

MRMutation was developed as a companion system to MRMPath, primarily because it involves several processing features that have been described for proteins (either cut and paste, or a protein from EXPASY, or proteins in a pathway stored in KEGG). MRMutation is available at the same resource that houses MRMPath. A user can deploy the software by entering a protein as its EXPASY Accession Number or by a descriptor for the protein, for example, TP53 human. In the latter case, the software accesses the EXPASY UniProt page for this entry. 

**Figure 5:**
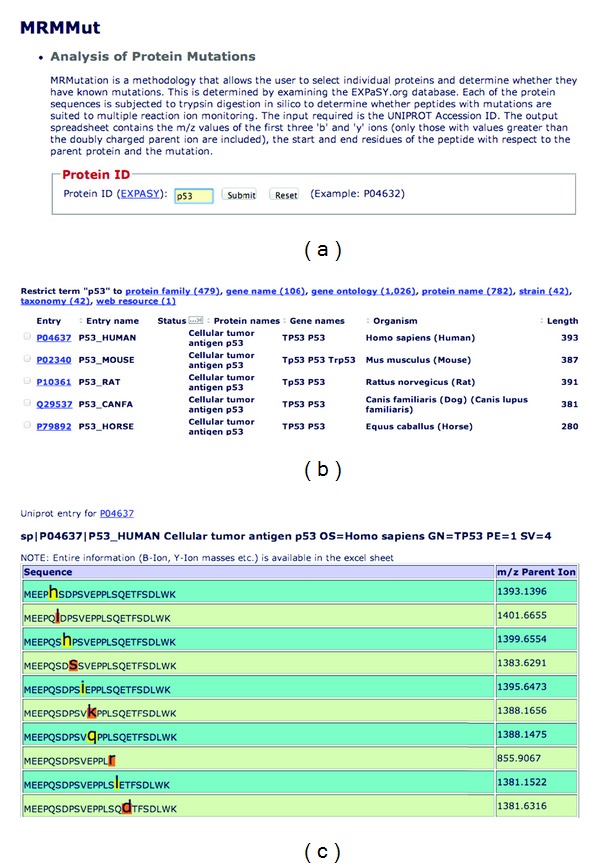
(a) The user interface for MRMutation. A user can input a free text search of the Accession ID of a UniProt entry. (b) The results (truncated) of a search in the interface identified records with the keyword “p53.” (c) Clicking the first link results in the creation of a tryptic digest of the protein identified through Accession ID P04637. The mutated amino acid residues are highlighted in the tryptic peptide sequence, along with the *m/z* of the parent peptide ion.

The user must select the appropriate protein record. Protein accession numbers with a “P” as the first character are the most informative about the known mutations of the protein. The unique identifiers for each of these mutations are then retrieved and the information for the protein is processed. Processing involves subjecting each protein (obtained as the protein's FASTA formatted sequence) to a tryptic digest. This is to determine whether peptides with mutations are suited to multiple reaction ion monitoring. An output similar to the one for each protein in MRMPath is produced, except that it only contains the peptides that contain mutations.  Figure 5 illustrates this table, where the mutated amino acid residue is highlighted. A table is generated that lists b- and y-ions whose masses are greater than the parent peptide ion *m/z* ratio. In addition, just as for results in MRMPath, the results of MRMutation are also available for download in a Microsoft Excel spreadsheet.

## 3. Discussion

The success of proteomics requires the development of informatics tools to enable the investigator to design targeted mass spectrometry experiments that answer specific biological hypotheses. Because of the immense amount of information involved, it has become important to create methods whereby biologists, in addition to mass spectrometry experts/operators, can contribute to the process. In the present study, MRMPath and MRMutation have successfully been put into practice to empower biologists to find those parts of a protein's sequence that are suitable for MRM analysis. These programs are also valuable to the mass spectrometry expert. 

The value of the approach taken in creating MRMPath is driven by the extensiveness of the information available in the KEGG database. While manually searching for information on a single protein in this database is feasible, when confronted by 20–30 members of an entire pathway, it became obvious that an automated approach was necessary. MRMPath allows the investigator to select the pathway and the species of interest and then uses a data mining approach to filter information that is associated with each protein. Once captured, the protein sequence information is processed on local computers that automatically “cut” the protein into smaller peptide sequences obeying the biochemical rules set by the protease that would be used. Peptide analysis using the MRM approach is more specific for peptides that have seven or more amino acid residues. On the other hand much larger peptides (>25 amino acids) are harder to detect in most current mass spectrometers. The MRMPath software filters the peptides from a given protein to create a list of those that have 7–25 amino acids. There are many posttranslational modifications to proteins (and hence peptides) in biological and pathologic systems. Some of these, particularly oxidations, can occur after the sample has been taken and while it is being processed, in preparation for analysis. The sulfur atoms in cysteine and methionine residues are particularly prone to this—in the case of cysteine, many investigators block its free sulfhydryl group with an alkylating agent prior to analysis. Since controlling this oxidation is difficult and variable, MRMPath automatically filters out peptides that contain cysteine or methionine residues.

MRMPath software takes each filtered peptide and calculates the *m/z* of its precursor (molecular) ion and the product b- and y-ions. The latter are restricted to those that have values (singly charged) that are larger than the *m/z *of doubly charged precursor ion. The higher *m/z* values ensure that a singly charged precursor ion cannot contribute to the analysis of the doubly charged peptide. 

It is important to verify the specificity of the peptide as a surrogate for the parent protein. Although a BLAST search could be done manually, MRMPath makes it a simple, clickable action. Indeed, the user can click just once to carry out a BLAST search on all peptides from a protein, although it may take several minutes for the BLAST search to be completed. The result of the MRMPath BLAST search may reveal that there is only one protein record that matches the peptide sequence, or it may indicate that there are multiple protein records. The latter may nonetheless be all the same protein since the NCBI database has many duplicate records for each protein. To assist the investigator, MRMPath-generated BLAST table of results contains a link to the full protein record. It should be noted that although MRMPath in combination with the BLAST search may indicate that a protein is specific in alphabetic space, the MRM-MS analysis is carried out in mass space and therefore the possibility remains of peptides with similar sequences that match the *m/z* of the precursor ion and the selected product ion. This would occur when a peptide had the same amino acids (i.e., the same molecular weight), but in a different order. In that case, there is value in obtaining the whole mass spectrum of product ions to verify the identity of the peptide. Although this is not easily obtained on a triple quadrupole (qqq) mass spectrometer, high sensitivity Qq-TOF mass spectrometers can provide this information.

As biomedical science moves into more and more use of DNA deep sequencing methods based on direct sequencing rather than hybridization to “known” sequences, it is becoming apparent that there is far more sequence variation in genes and hence proteins than previously realized. For some genes, the variation in sequence can occur between tissues in the same person. While germ-line DNA information is copied faithfully from parents to their children with little error, somatic tissues can have >1500 mutations in the whole genome [7]. This suggests that the so-called canonical sequence of a gene or protein may be subject to more variation than hitherto. Because of their potential involvement in disease, certain genes/proteins have been subject to considerable attention. One of these is the human protein p53, a regulator of the G_1_/S cell cycle checkpoint that is associated with many cancers.

 MRMutation allows an investigator to capture known information about mutations for a specific protein. It data mines the UniProtKB/SwissProt database, part of the EXPASY suite of programs. The investigator can either provide the protein accession number if they already know it, or they can describe the protein (e.g., “human” and “p53” would be the search terms). In the latter case, a table of proteins normally generated by the EXPASY software appears. For both, it is best to select the protein record that starts with a “P” since these records contain a compilation of all the known mutations and greatly facilitate the recovery of the required information. In the case of human p53 (P04637), there are currently (as of the preparation of this paper) mutations that lead to 1248 peptides that are different from those in wild-type human p53. At certain residue positions in this 393 amino acid protein, there are more than 10 different amino acids. For the human low-density lipoprotein receptor (P01130) there are 129 mutated peptides, whereas for NADP-dependent isocitrate dehydrogenase only two have been described. In contrast, pyruvate kinase (P30613) has 94 peptide mutations. 

 While there is some overlap between the utilities provided by MRMPath and Skyline software, there are also some distinct and significant differences. The principal ones are that MRMPath leverages the results of pathway analysis and is Internet browser driven. While Skyline provides detailed analysis of mass spectrometric data that is more extensive than MRMPath, its primary input is the output of a mass spectrometry experiment, namely, the DDA (data-dependent acquisition) file and therefore is not in the domain of a biologist. Skyline is capable of processing the results of and/or data from several commercial vendors. However, Skyline is available as a standalone system that only works on the Windows operating system. Furthermore, it has to be downloaded and installed. This is an advantage for those who wish to use its tools privately and offline.

From a bioinformatics and software development standpoint, the novel aspects of MRMPath and MRMutation are advancement of the notion of interoperability [8] in the realm of proteomics [9]. Interoperability is defined as the automated exchange of knowledge and data between resources that are repositories of heterogeneous (or heterogeneously stored) information. Interoperability has seen a significant rise that keeps up with the burgeoning information available online. Interoperability seeks to create a platform for information exchange while precluding the need to recreate information that is already available at the different resource. 

MRMPath and MRMutation programs access the resources, EXPASY and KEGG. The software development notions employed here are innovative because it involves access to and manipulation of information available online to better serve the users of the MRM resources. Use of MRMPath and MRMutation avoids the need to transfer and store all the protein information (from EXPASY) or all the pathway protein information (from KEGG) on local servers since the stored information would have to be continually updated. In addition, use of a dynamic mode of accessing BLAST avoids the storage of a local BLAST server. 

 The methods discussed in this paper are easily extensible to applications in other domains [10]. In MRMPath, the web page related to a biological pathway is downloaded and the URLs of the links therein are manipulated so that MRMPath can be deployed. The KEGG pathway downloaded by the investigator is a single webpage without additional burdens being placed on the KEGG servers. All processing is done at the UAB servers. MRMPath also represents a significant boon to mass spectrometrists, who wish to obtain surrogate peptides for proteins in any pathway in the KEGG databases.

For MRMutation, if the investigator enters a UniProtKB Accession Identifier in the text field, the URL that directs the browser to the full protein record in UniProt is modified so as to extract only the FASTA formatted protein sequence of the mutant, which is then subject to further processing. On the other hand, if a protein name is entered, MRMutation will access all the UniProt protein records that include the name. The expert user must then select the appropriate record for processing by MRMutation.

The value of MRMPath and MRMutation is that they leverage existing information (without having to recreate it). There is, on the other hand, the practical matter of the use of bioinformatics-based methodologies to rapidly and efficaciously process biological knowledge (particularly knowledge that is stored remotely and heterogeneously). Performing the same manually would be overwhelming from the standpoints of efficiency, accuracy of information processed and results obtained, and the time spent. 

MRMPath and MRMutation serve researchers over a range of biomedical domains. These include anybody with a proteomics-based interest in any pathway that is currently stored in KEGG. From the time an investigator enters a protein sequence into MRMPath (in one of the three ways discussed in Section 2), results will be obtained in a matter of a few seconds; the only barriers are the time taken for BLAST results. If an investigator wishes to perform MRMPath's task manually, he or she would have to access KEGG, chose a path for a species, click on the link for a specific protein, click on the FASTA formatted file for that protein, and download the FASTA file; depending on the choice of enzyme, he or she would have to create peptide fragments, filter them according to the specifications for additional processing, manually calculate y- and b-ions, take each peptide fragment and enter it into a BLAST search, and then process the final results. It is more than likely that manually performing all the steps that MRMPath would complete in a few seconds would take a few hours. If the same procedure was to be carried out for all the proteins in a pathway for a species, it would take several man-days of work, not accounting for fatigue and the consequent errors. For MRMutation, processing of proteomic data is even more efficient. 

 MRMPath and MRMutation are therefore an advantageous not just from the developmental standpoint, of an interoperability-based software, but also for their simplicity of use and significant advantage over manually accomplishing the same task. These software are freely accessible and need not be downloaded and installed. The only requirements are an Internet browser; hence, the systems are platform independent. All the processing takes place on the server side, and the graphical user interface for querying the system and the results are available instantly and dynamically on the same browser MRMPath and MRMutation can be freely accessed at http://tmpl.uab.edu/MRMPath/.

## 4. Conclusion

In summary, MRMPath and MRMutation are bioinformatics tools that will have great value in the design of experiments in quantitative proteomics, particularly in the analysis of biomarkers. 

## References

[1] Hillenkamp F, Karas M (1990). Mass spectrometry of peptides and proteins by matrix-assisted ultraviolet laser desorption/ionization. *Methods in Enzymology*.

[2] Fenn JB, Mann M, Meng CK, Wong SF, Whitehouse CM (1989). Electrospray ionization for mass spectrometry of large biomolecules. *Science*.

[3] Diz AP, Carvajal-Rodríguez A, Skibinski DOF (2011). Multiple hypothesis testing in proteomics: a strategy for experimental work. *Molecular and Cellular Proteomics*.

[4] Ransohoff DF (2010). Proteomics research to discover markers: what can we learn from netflix?. *Clinical Chemistry*.

[5] Gerber SA, Rush J, Stemman O, Kirschner MW, Gygi SP (2003). Absolute quantification of proteins and phosphoproteins from cell lysates by tandem MS. *Proceedings of the National Academy of Sciences of the United States of America*.

[6] http://www.nist.gov/pml/data/comp.cfm/.

[7] Conrad DF, Keebler JEM, Depristo MA (2011). Variation in genome-wide mutation rates within and between human families. *Nature Genetics*.

[8] Buetow KH (2005). Cyberinfrastructure: empowering a "third way" in biomedical research. *Science*.

[9] Cannataro M (2008). Computational proteomics: management and analysis of proteomics data. *Briefings in Bioinformatics*.

[10] Litwin W, Mark L, Roussopoulos N (1990). Interoperability of multiple autonomous databases. *Computing surveys*.

